# Celebrating 50 years since Lewontin's apportionment of human diversity

**DOI:** 10.1098/rstb.2020.0405

**Published:** 2022-06-06

**Authors:** Michael D. Edge, Sohini Ramachandran, Noah A. Rosenberg

**Affiliations:** ^1^ Department of Quantitative and Computational Biology, University of Southern California, Los Angeles, CA 90089, USA; ^2^ Department of Ecology and Evolutionary Biology, Brown University, Providence, RI 02912, USA; ^3^ Department of Biology, Stanford University, Stanford, CA 94305, USA

**Keywords:** genetic diversity, human evolution, population genetics

## 1. Introduction

‘85% of human genetic variation resides within populations'—Richard Lewontin's result and sound bite, tracing to his 1972 article ‘The apportionment of human diversity’ [1], have become indispensable to descriptions of worldwide human genetic variation. In addition to providing a technical advance for the field of human population genetics, the article provides a shorthand for the understanding of human genetic unity and an important response to the misappropriation of descriptions of human biological variation in support of racism.

This special issue brings together a collection of papers in recognition of the 50th anniversary of Lewontin's 1972 paper. With Lewontin's passing on 4 July 2021 at the age of 92 while this special issue was being assembled, the importance of the paper in the vast oeuvre of a giant in evolution, genetics and public understanding of science has become all the more apparent. The contributions in the special issue investigate the background, legacy and ongoing salience of ‘The apportionment of human diversity’. They consider the paper's scientific contribution and broader social relevance, also examining it in relation to some of Lewontin's other writings.

## 2. Lewontin's 1972 paper

Fifty years later, Lewontin's paper remains a lucid and stimulating account of an attempt to answer a basic question about human genetic variation: at a ‘typical’ genetic locus, how does the amount of genetic variation *within* populations compare with the amount of genetic variation *between* populations?

Lewontin's description of his approach is, as summarized by Novembre [2], admirably transparent, with a forthright description of the choices made with respect to the genetic loci, populations and statistical approach, as well as the likely effect of these choices on the results. Lewontin analysed data from 17 genetic ‘systems,’ protein variations assayed by immunological or electrophoretic methods. Although there was considerable uncertainty regarding the genetics underlying the observed protein variation, such data represented the best information available on genetic variation at the time.

Lewontin's choice of populations for testing a racial model of human variation was constrained in part by data availability, but beyond that, his discussion highlights the non-obvious decisions involved in weighting the populations and organizing them into ‘races.’ Lewontin's division considers seven groups, largely on the basis of racial ideas typical for the time. The list of populations and races in his Table 2 is perhaps the most obviously dated part of the paper, a reminder of the ways in which population labels and categorizations can frequently change.

Finally, Lewontin presents a statistical approach to partitioning diversity on the basis of a thoughtful discussion of desiderata for any diversity measure. He chooses an approach grounded in Shannon entropy that satisfies the required criteria. Lewontin's proportions of diversity among populations and ‘races’ can be viewed as entropy-based analogues of heterozygosity-based *F*_ST_-style statistics [3,4] which have since dominated approaches to the partitioning of genetic diversity.

With the table set, Lewontin serves the main dish, estimating that at an average genetic locus, 85.4% of the total diversity of the human species is within populations, 8.3% is among populations but within races and 6.3% is among races. He describes the finding with an enthusiasm unusual in scientific papers [1, p. 396]: ‘The results are quite remarkable …. Less than 15% of all human diversity is accounted for by differences between human groups!’ The fact that most genetic diversity lies within populations rather than between them is surprising if one takes observations of racialized physical traits such as skin colour to be representative of typical patterns of genetic diversity and divergence.

Lewontin's style shifts in transitioning to the paper's two final paragraphs, which provide his interpretation of the results [1, p. 397]:

It is clear that our perception of relatively large differences between human races and subgroups, as compared to the variation within these groups, is indeed a biased perception and that, based on randonly [*sic*] chosen genetic differences, human races and populations are remarkably similar to each other, with the largest part by far of human variation being accounted for by the differences between individuals.

Human racial classifcation [*sic*] is of no social value and is positively destructive of social and human relations. Since such racial classification is now seen to be of virtually no genetic or taxonomic significance either, no justification can be offered for its continuance.

The legacy of Lewontin's paper would be profoundly shaped by these concluding sentences.

## 3. The papers in the special issue

### (a) Context and impact of ‘The apportionment of human diversity’

Three articles in the special issue focus on the context and impact of Lewontin's 1972 paper. As described by Novembre [2], Lewontin framed his variance-partitioning question in terms of a larger discussion between perspectives then termed (e.g. [5]) the ‘classical’ school (typified by Hermann Muller) and the ‘balance’ school (typified by Lewontin's PhD mentor, Theodosius Dobzhansky), based on their perspectives about the role of natural selection in human evolution. Under the classical view, which held that heterozygosity would be rare, the apparent physical differences between people indigenous to geographically distant regions might be expected to represent a large fraction of the total variation. According to the balance school, which expected higher heterozygosity, such differences might be expected to represent a smaller proportion of total variation. This setup was natural given Lewontin's then-recent work in assaying genetic variation, in which he found patterns more aligned with the Dobzhanskian prediction.

Novembre [2] discusses the endurance of the result: as human population genetics developed beyond the 17 markers used by Lewontin, subsequent studies obtained similar findings. Given the small number of loci that Lewontin analysed, the possibility that those immunological and enzymatic loci were not representative, the vagaries of sampling that resulted in his dataset, the uncertainties regarding the weighting of populations and their organization into higher-level groups, and the use of a custom diversity measure that differs somewhat from more typical methods in population genetics, it is perhaps remarkable that Lewontin's results have replicated so robustly. Indeed, studies that have confirmed Lewontin's result have used other types and larger numbers of genetic loci, distinct populations and schemes for organizing them, and different statistical frameworks. Novembre also analyses and seeks to reconcile criticisms that have emerged regarding Lewontin's study, and he provides suggestions about teaching its result.

Shen & Feldman [6] discuss how Lewontin's study emerged not only from the connection to the ‘classical’ and ‘balance’ argument in evolutionary biology, but also from ongoing discussions of race and genetics that flared after the publication of inflammatory claims prior to Lewontin's study. They then look ahead to Lewontin's subsequent work, tracing the paper as an element of Lewontin's career-long efforts to argue against biological-determinist views of human traits and incorrect or unsupported attributions of complex phenotypic differences among human populations to genetic differences among populations. They reflect on the continuing relevance of Lewontin's work against racism, biological determinism, and adaptationism in light of the re-emergence of such ideas.

Carlson & Harris [7] discuss the bibliometric impact of the paper as a touchstone in the development of an understanding of human genetic variation as not racially structured. They discuss how the force of Lewontin's rejection of human racial classification set his paper apart from similar analyses that appeared around the same time. They comment on ways in which Lewontin's closing statement was adopted by scholars outside genetics, becoming the focus of continuing discussion. Carlson & Harris also examine the study's importance as a major contribution in the statistical and empirical study of human population genetics, areas that grew rapidly with genetic technology that developed in the 1990s and beyond.

### (b) Statistical analysis of population-genetic data

Lewontin's 1972 paper [1] is memorable as a technical milestone in population-genetic data analysis. Several articles in the issue represent entries in the lineage of Lewontin's paper as a work of statistical population genetics, with clear connections to issues present in Lewontin's study. Indeed, statistical issues to which it connects, on genetic diversity statistics, population classification, and natural selection in human evolution, continue to be fundamental lines of inquiry in human population genetics.

Three of the articles focus on the mathematical and statistical properties of summary statistics for patterns of genetic variation, considering approaches similar to that of Lewontin [1]. Examining *F*_ST_ statistics that have overtaken Lewontin's entropy approach to variance partitioning, Alcala & Rosenberg [8] study the mathematical constraints that are imposed on *F*_ST_ by the number of populations that appear in a computation and the frequency of the allele that is most frequent across the populations. In an example application, they show that the constraints can explain peculiar observations of *F*_ST_ in data involving chimpanzees. Interestingly, similar attention to constraints on summary statistics and dependences of these constraints on allele frequencies were anticipated by Lewontin in his *D*′ normalization [9] of his linkage disequilibrium measure *D* [10] and his later comments on features of linkage disequilibrium statistics [11]. As Alcala & Rosenberg [8] discuss, mathematical properties of *F*_ST_ in part explain why studies after that of Lewontin [1] have varied slightly in their estimates of variance components, since the studies are estimating quantities that differ on the basis of the number of population groupings and the allele-frequency constraints on different marker types.

A second important approach to *F*_ST_ considers the behaviour of *F*_ST_ estimators under specific evolutionary models. Guerra & Nielsen [12] perform a study in this tradition. After arriving at expressions for the covariance of pairwise coalescence times under a general framework, they consider the behaviour of estimators of Slatkin's formulation of *F*_ST_ [13], which casts *F*_ST_ in terms of a ratio involving coalescence times for pairs of lineages randomly drawn from the same subpopulation versus pairs of lineages randomly drawn from the total population. Among other results, Guerra & Nielsen show that a commonly used estimator for single-locus *F*_ST_ is biased as an estimator of Slatkin's *F*_ST_, providing an argument in favour of the ‘ratio of averages’ approach to *F*_ST_ estimation, in which estimates of a numerator and a denominator are pooled across loci, and a ratio of these quantities is adopted as the estimate (e.g. [14]).

Peter [15] investigates properties of a collection of statistics that, like *F*_ST_, also consider sums of squares of allele frequencies. Peter considers the relationship between principal component analysis (PCA) and the widely used ‘*F*-statistics’ *F*_2_, *F*_3_ and *F*_4_ of Patterson *et al.* [16], which have been particularly important in the analysis of ancient DNA. After providing an exposition of *F*-statistics and their interpretation in a geometric framework developed by Oteo-Garcia & Oteo [17], Peter considers the geometry of *F*-statistics in a principal-component (PC) space formed by analysis of population-level allele frequencies. In this setting, *F*-statistics and the relative positions of populations on a PC plot can mutually constrain each other. For example, pairs of populations with low *F*_2_ are constrained to be close together on a PC plot, and *F*_3_ is constrained to be positive if a putative admixed population is outside a circle defined in relation to the putative source populations (with a radius determined by the genetic distance between the putative sources) on any two-dimensional PC plot. These results provide an illuminating view of the meaning of both *F*-statistics and PCA.

In a modelling study, Yair & Coop [18] investigate the process of population differentiation under stabilizing selection, a process that plausibly underlies the evolution of many complex traits. Empirical results such as Lewontin's have established the relatively low locus-by-locus-level genetic differentiation of human populations, and long-standing results from quantitative genetics predict that under neutral evolution, the degree of population differentiation in a heritable trait is expected to mirror population differentiation at a typical locus. Under a model of stabilizing selection in which several populations share a fitness ‘optimum’ for the value of a trait, the trait differentiation is typically expected to be smaller than predicted by neutral models. Yair & Coop find that, counterintuitively, although stabilizing selection with a shared optimum decreases population differentiation on a trait, it also can cause population-mean polygenic score values to appear more differentiated than they would under neutrality. Stabilizing selection pushes the frequencies of alleles that affect the trait quickly toward 0 or 1, leading to rapid turnover of trait-associated alleles in each population. Their simulation studies are accompanied by theoretical predictions regarding population differentiation at trait-underlying loci and the phenotypic variance explained by ancestral polymorphisms over time. These results add to a growing set of cautions about the interpretation of population-mean polygenic scores, and they also provide one explanation for the decreased predictive value of polygenic scores in populations other than the ones in which effect sizes were estimated.

Four studies in the issue focus on empirical aspects of population-genetic variation. Witt *et al.* [19] perform analyses similar to Lewontin's apportionment computation, but they focus on the geographical distribution of genetic variants inherited from archaic Neanderthal and Denisovan populations. They find that the comparative extent of archaic variation in East Asian, European and South Asian modern populations depends on whether the amount of archaic variation is tabulated within a single genome or in the collection of genomes from a population. In their analysis, among the three groups, the South Asian group possesses more archaic alleles not found in the other groups; however, at the individual level, the number of archaic variants is greatest in East Asian genomes. These contrasting results in different data summaries are reminiscent of the way in which different results are seen in conceptually distinct summaries of the geographical distribution of global human variation [20,21], including Lewontin's apportionment. Witt *et al.* anticipate the similar interpretive challenge of reconciling multiple summaries in the relatively new study of archaic variation.

Aylward *et al.* [22] illustrate the links that exist between studies of human population structure and analogous population structure computations in a non-human system. Focusing on a species of intense interest, Aylward *et al.* review studies of the population structure of wild tigers, demonstrating the progression of past studies through genetic markers similar to those used in human populations, employing summary statistics and population clustering methods common in human population-genetic investigations. The paper illustrates the connections in statistical and empirical methods between human population genetics and related population-genetic studies in conservation and molecular ecology. The authors note Lewontin's role in the development of these connections through his early work on allozyme variation [23,24].

Rodriguez-Rodriguez *et al.* [25] perform an empirical study of a set of modern human populations in relation to a historical event. In a genomic analysis of diverse populations focused on Mexico, they uncover a signal of Southeast Asian genetic admixture in Mexican populations in the coastal state of Guerrero. Examining the lengths of genetic segments that appear to represent ancestry from Southeast Asia, Rodriguez-Rodriguez *et al.* trace the signal to voyages that took place between Acapulco and The Philippines starting during the 1500s, surmising that these voyages carried sufficiently many people from The Philippines to Mexico to leave a genetic signal today. The study required enough markers to reveal a subtle pattern, illustrating the dramatic advance from Lewontin's coarse study of 17 markers to the much finer investigations of population structure and historical descent feasible using population-genetic data today.

Broadening outward to the phenomenon of human genetic admixture more generally, Gopalan *et al.* [26] review recent trends in demographic studies of the genetic history of admixed populations. As noted by Shen & Feldman [6], the classification system Lewontin sought to test did not have a clear role for genetic admixture, a phenomenon that has generated great interest in modern human population genetics. Gopalan *et al.* find that, as represented in the example of Rodriguez-Rodriguez *et al.* [25], advances in statistics and data produce a rich picture of genetic admixture in human populations around the world, deepening findings on admixture far beyond what was possible in 1972.

### (c) Practical problems in human genetics

Much of the discussion of ‘The apportionment of human diversity’ and its legacy has been focused on the abstract discussion of human genetic variation and its structure. However, for specific practical problems, some explicit or implicit understanding of human genetic structure is required. Three papers in the special issue consider ways of understanding human variation in applied contexts, illustrating differences among theories of human variation used in certain social settings.

Jobling [27] discusses forensic genetics, an important societal application of population-genetic ideas. Although allele-frequency differences among human populations tend to be small, Lewontin forcefully pointed out that they nonetheless introduce complications when one is trying to quantify the strength of evidence provided by a genetic match between a suspect and a crime-scene sample. In particular, he was concerned that the degree of evidence against a defendant might be overstated if the wrong allele-frequency model was used. As Jobling notes, the solution adopted by the forensics community did not satisfy Lewontin, but his early objections were an important influence on the field. Jobling reviews more recent developments at the intersection of forensic genetics and population structure, considering methods to gain information about the source of a crime-scene sample when there are no suspects and no matches in a database. Jobling argues that some of these efforts, such as attempts to infer biogeographical ancestry or physical appearance, end up emphasizing between-population components of variation rather than the larger within-population component, potentially creating a distorted impression of human genetic variation.

Kaplan & Fullerton [28] consider contemporary efforts to predict disease risk using polygenic scores in light of Lewontin's 1972 paper. As in forensic genetics, human genetic structure, despite generally low levels of differentiation, causes complications for polygenic scores. These complications arise both in their estimation, because confounding due to population stratification can lead to errors in estimating the scores, and, as discussed by Yair & Coop [18], in their application, as polygenic scores estimated in one population do not predict phenotypes as well when used in other populations. Kaplan & Fullerton draw out these and other tensions that arise in a setting of widespread health disparities and social patterns of inequity that correlate with aspects of human population structure. They arrive at a position sceptical of the promise of polygenic scores—even those based on samples diverse in ancestry—to understand or resolve health disparities in the absence of close attention to social context.

Maróstica *et al.* [29] examine human population structure in the setting of the human leucocyte antigen (HLA) and bone-marrow transplantation matching. In transplantation matching, the goal is to find donors with a sufficiently close genetic match to patients at HLA loci in order to prevent rejection of the transplanted tissue by the patient. Maróstica *et al.* comment that the HLA loci are extremely diverse across human populations, owing in part to balancing selection. For mathematical reasons (e.g. [8]), traditional population-structure statistics such as *F*_ST_ remain low for these loci, obscuring the global diversity. The pattern of HLA variation is such that close matches are most likely between people with similar ancestry. Because the transplantation process itself is organized in human societies, the interaction of the underlying population genetics with the variability of donor recruitment across populations can lead to significant differences in the possibility of identifying matching donors for a patient. Maróstica *et al.* discuss this problem in Brazil, particularly in the context of people of African descent, a group with high genetic variability and relatively low donor recruitment rates.

## 4. Lewontin's 1972 paper: a celebration

As discussed in particular by Carlson & Harris [7], Lewontin's paper is iconic, and its impact is far-reaching. This special issue is not the first to specifically commemorate it, as a chapter by Ruvolo & Seielstad [30] in a Lewontin festschrift volume gave a wide-ranging account of subsequent confirmations of its finding, the position of the paper in human evolutionary genetics more generally, and experiences of teaching Lewontin's result. Furthermore, during preparation of this special issue, we became aware of other ongoing commemorations.^1^

However, as noted by Novembre [2], Shen & Feldman [6] and Carlson & Harris [7], Lewontin's paper has also generated disagreements. For example, ignoring differences among populations can lead to challenges in addressing effects of genetic variation in biomedical problems. The genetic differences that do exist can be relevant in biomedical settings, in part because they can affect population-level differences in Mendelian disease allele frequencies, genetic risk factors for complex disease, transplantation matching probabilities and treatment responses (e.g. [28,29]). As discussed by Novembre [2], Lewontin's paper [1] has been referenced beyond the field of human evolutionary genetics as supporting an oversimplified view of human variation, in which population structure is completely ignored even in situations in which it might be relevant, such as matching problems in forensics or transplantation, where population variation in match probabilities affects the societal use of population-genetic computations [27,29].

Novembre [2] and Shen & Feldman [6] address a criticism of Lewontin's paper that emerged from this perception of the paper's role, concerning the relationship between variance partitioning and ancestry inference; this criticism is most closely associated with Edwards [38] and also appeared in an earlier instantiation [41]. Novembre [2] and Shen & Feldman [6] describe how two distinct questions can be posed about human genetic variation: (1) What are the magnitudes of the within-population and between-population variance components in a partition of human genetic variation? (2) Can the collection of genetic variants in an individual genome be used to infer the individual's genetic ancestry? As noted by Neel [42], Rosenberg [39] and Lewontin himself together with Feldman [43], Lewontin recognized the distinction between these questions, advocating for the primacy of the first for understanding the extent to which individual genotypes and phenotypes can be predicted from group membership. The title of Edwards's paper gives the name ‘Lewontin's fallacy’ to the claim that ancestry inference is not possible when the between-population variance component is small. Edwards is correct in identifying as erroneous the statistical reasoning he termed ‘Lewontin's fallacy’; however, as Novembre [2] and Shen & Feldman [6] point out, the error is one that Lewontin himself did not make. Further, Lewontin recognized the importance of genetic variation among populations in biomedical problems; indeed, Feldman & Lewontin [43] included a perspective on the value of genetic ancestry in such contexts.

In seeking to understand the factors that propelled Lewontin's paper to its present iconic status, Carlson & Harris [7] trace the paper's bibliometric trajectory, contrasting it with the contemporaneous work of Nei & Roychoudhury [44,45] that produced similar technical results but is not similarly celebrated. Carlson & Harris [7] argue that the widespread recognition of Lewontin's paper is partly situated in the comments in its final lines and Lewontin's strong stance against human racial classification on the basis of the 85% within-population variance result. However, as Novembre [2] notes, even if we disregard Edwards's exchange as targeted at a misunderstanding of Lewontin's paper rather than a genuine critique of Lewontin's argument, Lewontin's brief concluding interpretation is incomplete. A more comprehensive view can be found by studying the 1972 paper together with Lewontin's other writings, as in Shen & Feldman [6].

Examining Lewontin's work of the 1970s, Shen & Feldman [6] explain that the theory of race that Lewontin was rejecting at the end of his paper was a theory of essentialized race, in which individual members of a race possess group-specific traits and trait values. As they discuss, under such a theory, the membership of an individual in a race is strongly predictive of the individual's traits. Lewontin's result, in which, for a typical genetic locus, the within-population variance component far exceeds the between-population variance component, stands in contrast with such an essentialized view, suggesting that traits for which group membership predicts individual trait values are unusual rather than typical of traits in general. Indeed, such a conclusion follows almost immediately for single-locus phenotypes, if Lewontin's results can be taken as representative of the relevant loci. For complex traits, there are more possibilities, but subsequent phenotypic modelling under neutral models argues that a high within-population variance component for typical genotypes suggests a similarly high within-population variance component for many phenotypes—and the within-population component will be even higher on average if populations experience stabilizing selection that selects for the same optimal phenotypic value (reviewed by Yair & Coop [18]).

Although Lewontin's 1972 paper demonstrates that the essentialized racial theory is unsupported genetically, philosophical discussion since the paper has asked if some other theory of biological race with a weaker predictive ability could accommodate Lewontin's result (e.g. [46]). However, whether or not this philosophical discussion can find such a ‘deflationary’ theory, it is the result itself rather than the accompanying theory that would remain most directly relevant for understanding human variation; any claims deriving from such a theory of race would need to be compatible with the results of Lewontin [1] and subsequent similar results that have been repeatedly observed.

Shen & Feldman [6] ask us to consider the 1972 paper in relation to some of Lewontin's other work of the 1970s and 1980s on hereditarian biological racism, the perspective that attributes group differences in phenotypes to unchangeable heritable qualities that differ between groups, and ascribes value to those group differences. They argue that, then and now, hereditarian biological racism rests on three claims: (1) the claim that human diversity is structured racially, with pronounced genetic differences between ‘races’; (2) the claim that differences across populations in distributions of meaningful complex traits trace straightforwardly to genetic differences between populations; (3) the claim that such differences in distribution have a simple basis in past adaptation of different populations to different environments. Lewontin's writings of the 1970s and 1980s explain the indefensibility of all three of these recurring claims, with the 1972 paper focused on the first of the three.

Hence, we can view Lewontin's 1972 paper as a landmark paper not only for its arguments against a theory of biological race prominent at the time of its publication, but for its role as a key step in a broader argument against hereditarian biological racism. Its iconic contribution lies in the way in which its statistical finding itself, rather than Lewontin's comments in the final lines of the paper, undermines the potential for racist theories to find support in genetic variation—as has been revealed through decades of the subsequent literature of the field, including subsequent work by Lewontin himself. The similarity of biological-racist claims in the time of Lewontin's paper and in the present are apparent in the references tabulated by Carlson & Harris [7]. As noted by Shen & Feldman [6], Lewontin's writings continue to provide a guide for understanding why such claims are unsupported by the science of genetics, with the 1972 paper and its legacy as an important component of that understanding. We are pleased to offer a celebration of this paper as a milestone in population genetics and our shared understanding of the human species.

## Data Availability

This article has no additional data.

## Endnote

1 Lewontin's paper [1] is discussed in detail in an essay by Roseman [31] and in chapters by Edwards [32], Gannett [33], Hochman [34], Kaplan [35] and Winther [36] in an edited volume [37]. Like Novembre [2] and Shen & Feldman [6], Winther [36] gives a detailed account of the construction of Lewontin's paper. Winther attempts to reproduce Lewontin's calculations, finding a number of minor calculation errors; according to Winther's calculations, Lewontin's own analysis should have produced 86% rather than 85% for the within-population variance component. Gannett [33] situates Lewontin's paper and the critique by Edwards [38] in a discussion of human variation, classification, and race over the long term; Kaplan [35] considers these papers in relation to current discussions of race and racism. Roseman [31] gives a detailed analysis of Edwards's critique, and, like Novembre [2] and Shen & Feldman [6], argues that Edwards's term ‘Lewontin's fallacy’ is unwarranted. Like Carlson & Harris [7], Roseman discusses abuses of the existence of this term to misleadingly appear to invalidate Lewontin's result, when, as discussed by Novembre [2], Shen & Feldman [6], and also Rosenberg [39] and Hochman [34,40], it does not; Carlson & Harris [7] memorably discuss this abuse as use of a ‘rhetorical cudgel in an attempt to dismiss an opposing argument as logically invalid'. For his own part, choosing not to further discuss the content of his 2003 critique, Edwards [32] gives a personal account of its origin; Edwards also finds a remarkable 1839 quotation from the well-travelled Robert FitzRoy, captain of the HMS *Beagle* on its famous 1831–1836 voyage, in which FitzRoy expressed the same sentiment as Lewontin [1]: ‘The conclusion to which I have been obliged to come is – that there is far less difference between most nations, or tribes (selecting any two for the comparison), than exists between two individuals who might be chosen out of either one of those nations or tribes; colour and hair alone excepted.’
